# An Update on Psychoactive Substances: Pharmacology and Toxicology Issues

**DOI:** 10.3390/ph16081177

**Published:** 2023-08-18

**Authors:** Stefania Chiappini, Fabrizio Schifano

**Affiliations:** 1 School of Medicine, UniCamillus International Medical School University, Via di S. Alessandro 8, 00131 Rome, Italy; 2 Psychopharmacology, Drug Misuse and Novel Psychoactive Substances Research Unit, School of Life and Medical Sciences, University of Hertfordshire, Hatfield AL10 9EU, UK; f.schifano@herts.ac.uk

This Special Issue, titled “Psychoactive Substances: Pharmacology and Toxicology”, aims to provide an up-to-date overview of the pharmacology, clinical information, and toxicology of psychotropics, as well as the effects associated with their intake. The increasing prevalence of psychoactive substances, both illicit and prescribed [1,2], demands our attention towards these crucial, yet often overlooked, aspects of public health. Psychoactive substances are diverse, ranging from cannabinoids, opioids, stimulants, and hallucinogens to prescription medications, such as antidepressants and anxiolytics. By interacting with the central nervous system (CNS), primarily targeting specific receptors and neurotransmitter systems, they alter brain function, resulting in changes in perception, mood, consciousness, cognition, or behaviour. Indeed, despite the current levels of knowledge and significant related morbidity, our understanding of the consequences of their use/misuse/abuse is limited [1,3].

The six published articles highlight different aspects of the pharmacology and toxicology of several psychotropics; most of them are represented by opiates/opioids, e.g., tramadol [4,5], tapentadol [4], and fentanyl [6], but other categories here mentioned are selective serotonin reuptake inhibitor (SSRI) antidepressants [7], new psychoactive substances (NPSs) known as cathinones [8], and the hallucinogen drug psilocybin [9].

The first paper [4] aimed to comparatively assess the rewarding and reinforcing properties of the synthetic opioids tramadol and tapentadol, at a therapeutic dose; the conditioned place preference (CPP) assay, a non-clinical animal paradigm of addiction potential in rats, was used. Both opioids showed short-term reinforcing properties; however, only tramadol showed the potential to induce drug memory and the incubation of craving several days after the last drug administration, suggesting that tapentadol has a lower propensity to induce dependence than tramadol.

The second study [5] investigated the mechanisms of tramadol-attributed seizures in rats, which have been hypothesized to involve both serotoninergic and non-serotoninergic, e.g., γ-aminobutyric acid-(GABA)ergic, histaminergic, dopaminergic, and opiatergic pathways. According to the results of the study, tramadol seizures were only prevented by diazepam, a positive allosteric modulator of GABAA receptor, suggesting that tramadol-induced seizures result from tramadol’s interaction with the GABAA receptor, involving a non-competitive mechanism at the benzodiazepine-binding site and that a strategy primarily relying on benzodiazepines may be appropriate in the management of tramadol-induced seizures.

Consistent with the need to investigate not only psychiatric but also medical issues related to the use of opioids, in a review from Chamoun et al. [6], the medical risks of opioids, including new synthetic opioids, were described. According to the authors, central nervous system depression resulting from opioid overdose and/or misuse of fentanyl analogues is clinically characterized by the onset of consciousness impairment, pinpoint miosis, and bradypnea. However, in contrast with what is observed with most opioids, the neurorespiratory toxicity of fentanyl and its analogues may be characterized by rapid-onset thoracic rigidity, possibly related to the activation of noradrenergic and glutamatergic coerulospinal neurons and dopaminergic basal ganglia neurons, contributing to an increase in the risk of death in the absence of immediate life support. Indeed, due to the high fentanyl levels of affinity for the mu-opioid receptor, the need to administer naloxone dosages higher/much higher than those usually required in morphine/heroin overdose to reverse the neuro-respiratory depression was identified here.

The potential for the abuse of medications that are controlled substances is well known; however, the abuse/dependence of certain noncontrolled prescription drugs and over-the-counter medications may also occur. During the past twenty years, this phenomenon, known as ‘pharming’ [10,11,12,13,14], has been increasingly reported. In relation to this issue, Chiappini et al. [7], in their study, assessed the available pharmacovigilance misuse/abuse/dependence/withdrawal signals relating to the SSRIs citalopram, escitalopram, paroxetine, fluoxetine, and sertraline. In doing so, they analysed both the EudraVigilance (EV) and Food and Drug Administration (FDA) Adverse Events Reporting System (FAERS) datasets. The EV misuse-/abuse-related adverse drug reactions (ADRs) were mostly recorded for citalopram, fluoxetine, and sertraline; conversely, dependence was mostly associated with paroxetine and withdrawal to escitalopram. Similarly, in the FAERS dataset, dependence-/withdrawal-related signals were more frequently reported for paroxetine, possibly due to its specific pharmacokinetic and pharmacodynamic properties.

Over the last decade, the consumption of the NPS synthetic cathinones for recreational purposes has increased in popularity, although it is associated with several toxic effects, even fatalities [14,15]. As all NPSs in general, synthetic cathinones are not controlled by international drug control agreements, although some of them may be subject to national regulatory measures; cathinones are a group of stimulant substances related to cathinone, the main psychoactive substance in khat (*Catha edulis*) [15]. They are marketed as ‘legal’ replacements to controlled stimulants, such as amphetamine, MDMA, and cocaine [16,17]. Among the most consumed cathinone derivatives in the United States of America and Europe are pentedrone and methylone. As all cathinone derivatives contain a stereogenic centre, their toxicokinetic and toxicodynamic characteristics can be different for each enantiomer. Indeed, the aim of the study from Silva et al. [8] was to assess the cytotoxic and metabolic profile of pentedrone and methylone enantiomers using physiologically relevant in vitro models. The hepatotoxicity of these compounds was observed in a concentration-dependent manner in human-stem-cell-derived hepatocyte-like cells (HLCs) cultured under 3D (3D-HLCs) and 2D (2D-HLCs) conditions. Methylone and pentedrone showed distinct and preferential metabolic routes for their enantiomers, resulting in the production of differentiated metabolites; R-(+)-methylone and R-(−)-pentedrone were the most metabolized enantiomers. Enantioselectivity in cytotoxicity for pentedrone was demonstrated.

Among psychedelic substances, psilocybin has been proposed as a promising transdiagnostic treatment strategy for a wide range of psychiatric disorders, including depression and anxiety disorders; recent results have shown that psychedelic-assisted/‘psycholytic’ psychotherapy may provide significant and lasting relief from depressive symptoms [18]. However, to date, several limitations of the published studies (e.g., small sample size, lack of blinding, limited follow-up, highly selected treatment populations) have been emphasized. Further confounding issues have included the diversity of practitioner experience in the different studies, the lack of standardized protocols, differences in legal status of psychedelics between the different countries, and a range of ethical concerns and potential psychological/psychopathological/medical adverse effects. In the opinion article published here [9], a number of hypotheses concerning the mechanism of action of psilocybin are described. However, at this stage, it is not entirely clear which signalling pathways are predictive of the therapeutic effect. Indeed, psilocybin acts as an agonist of the 5-hydroxytryptamine 2A receptor (5-HT2AR), which has been convincingly demonstrated as necessary for the occurrence of psychedelic drug effects and, specifically, for the propensity to induce visual hallucinations associated with psilocybin; this mechanism of action, however, may not be behind the occurrence of psilocybin for the antidepressant effects. The authors have summarized the clinical experience to date taking into account the efficacy and safety issues, the clinical challenges, and the limitations of previous studies; some suggestions for future studies have been provided here as well.

Overall, this Special Issue highlighted that scenarios relating to the vast range of prescribed and recreational psychoactive substances are multifaceted and complex. The knowledge of these molecules’ pharmacology and toxicology is integral to promoting public health and advancing medical science. Only with an enlightened approach can we harness their potential benefits whilst mitigating their inherent risks. In light of these diverse contributions, the importance of multidisciplinary teams using the expertise of laboratory researchers, clinicians, psychiatrists, and neurologists is undisputable. However, there are still many fundamental questions that remain unanswered, and these questions form the basis of a great future for this field. We strongly encourage a wide group of readers to draw inspiration from this Special Issue and develop new approaches to the study and treatment relating to psychotropics. Finally, the Guest Editors would like to sincerely thank all the authors and reviewers for their valuable contributions. 
